# Daily ingestion of catechin-rich beverage increases brown adipose tissue density and decreases extramyocellular lipids in healthy young women

**DOI:** 10.1186/s40064-016-3029-0

**Published:** 2016-08-18

**Authors:** Shinsuke Nirengi, Shiho Amagasa, Toshiyuki Homma, Takeshi Yoneshiro, Saori Matsumiya, Yuko Kurosawa, Naoki Sakane, Kumiko Ebi, Masayuki Saito, Takafumi Hamaoka

**Affiliations:** 1 Division of Preventive Medicine, Clinical Research Institute, National Hospital Organization Kyoto Medical Center, 1-1 Mukaihata-cho, Fukakusa, Fushimi-ku, Kyoto 612-8555 Japan; 2 Department of Preventive Medicine and Public Health, Tokyo Medical University, 6-1-1 Shinjuku, Shinjuku-ku, Tokyo 160-8402 Japan; 3 Faculty of Sports and Health Science, Daito Bunka University, 1-9-1 Takashimadaira, Itabashi-ku, Tokyo 175-8571 Japan; 4 Department of Biomedical Sciences, Graduate School of Veterinary Medicine, Hokkaido University, Kita 18, Nishi 9, Kita-ku, Sapporo, 060-0818 Japan; 5 Department of Food Science and Nutrition, Mukogawa Women’s University, 6-46, Ikebiraki-cho, Nishinomiya, 663-8558 Japan; 6 Department of Sports Medicine for Health Promotion Tokyo Medical University, 6-1-1 Shinjuku, Shinjuku-ku, Tokyo 160-8402 Japan; 7 Graduate School of Sport and Health Science, Ritsumeikan University, 1-1-1 Nojihigashi, Kusatsu, Shiga 525-8577 Japan; 8 Hokkaido University, Kita 8, Nishi 5, Kita-ku, Sapporo, 060-0808 Japan

**Keywords:** Near-infrared spectroscopy (NIRS), Noninvasive, Brown adipose tissue (BAT)

## Abstract

**Purpose:**

Brown adipose tissue (BAT) contributes to the regulation of non-shivering thermogenesis and adiposity. Increasing BAT has recently attracted much attention as a countermeasure to obesity. Animal studies have shown that prolonged catechin treatment increases uncoupling protein 1, a thermogenic protein in BAT. On the other hand, supportable evidence in human is lacking. Thus, the purpose of this study was to examine whether BAT increases after catechin ingestion in humans.

**Methods:**

Twenty-two healthy young women were given either a catechin-rich (540 mg/day; catechin) or placebo beverage every day for 12 weeks in a double-blind design. BAT density was measured using near-infrared time-resolved spectroscopy (NIR_TRS_), visceral fat area were measured using magnetic resonance imaging, extramyocellular lipids (EMCL) using proton magnetic resonance spectroscopy, and body fat mass using dual-energy X-ray absorptiometry scans.

**Results:**

BAT density was significantly increased (18.8 %), and EMCL was decreased (17.4 %) after the 12-week ingestion. There was a significant negative correlation between the changes in BAT density and those in EMCL (*r* = −0.66, *P* < 0.05). There were no notable changes in other parameters.

**Conclusions:**

In conclusion, prolonged ingestion of a catechin-rich beverage increases the BAT density in parallel with a decrease in EMCL.

## Background

Brown adipose tissue (BAT) produces heat during cold exposure and spontaneous overfeeding via upregulation of uncoupling protein 1 (UCP-1), whereas white adipose tissue stores excess energy as triglycerides in mammals (Cannon and Nedergaard 2004). In adult humans, metabolically active BAT has been identified in the supraclavicular and paraspinal regions in radionuclide studies using ^18^F-fluorodeoxyglucose positron emission tomography combined with computed tomography (FDG-PET/CT) (Cypess et al. 2009; Saito et al. 2009; van Marken Lichtenbelt et al. 2009; Virtanen et al. 2009). BAT is associated with cold-induced thermogenesis (Yoneshiro et al. 2013), glucose tolerance (Chondronikola et al. 2014; Hanssen et al. 2015; Lee et al. 2014; Matsushita et al. 2014), and lipid metabolism (Chondronikola et al. 2016) in humans as well as in animals. Therefore, an increase in the amount of BAT is expected to act as a countermeasure to obesity and obesity-related disease. However, due to the limitations of FDG-PET/CT, such as radiation exposure, it is difficult to conduct longitudinal intervention studies on the effects of an increase in the amount of BAT in humans.

Near-infrared time-resolved spectroscopy (NIR_TRS_) is a method for noninvasively determining total hemoglobin concentration [total-Hb], which reflects the capillaries vascularity in tissue (Hamaoka et al. 2007). Recently, the [total-Hb] under thermoneutral conditions was positively correlated with BAT activity (SUV_max_) estimated by FDG-PET/CT in the supraclavicular region, which potentially contains BAT deposits (*r* = 0.73) (Nirengi et al. 2015). Considering the abundant vascularity of BAT compared with that of other tissues, our results suggest that [total-Hb] estimated by NIR_TRS_ provides BAT density at the approximately 4-cm^3^ tissue (Nirengi et al. 2015). Quite recently, our new longitudinal study reported that the [total-Hb] increases with the increasing in the SUV_max_ evaluated by FDG-PET/CT during repeated thermogenic capsiate intake, which is known to increase BAT activity and mass. The amplitude of the increment of [total-Hb] (46.4 %) and SUV_max_ (48.8 %) is equivalent (Nirengi et al. 2016), thus confirming the validity of our measurements in longitudinal experimental setups.

Catechin is found in green tea, which has been habitually consumed in Asian countries for a long period of time (Nagao et al. 2009). Functional foods approved by US Food and Drug Administration (FDA) include those rich in catechin (Nagao et al. 2009). It has repeatedly reported that a single ingestion of green tea extract rich in catechins increases energy expenditure in humans (Dulloo et al. 2000; Gosselin and Haman 2013; Hursel and Westerterp-Plantenga 2013). Consistent with the acute thermogenic effect, daily ingestion of green tea catechins results in a small but significant reduction of body fatness (Nagao et al. 2005; Friedrich et al. 2011; Hursel et al. 2011).

Animals studies have also shown that 8 weeks of catechin intake upregulated expression of UCP-1 mRNA in BAT and decreased white adipose tissue mass compared with animals on a normal diet (Nomura et al. 2008). Choo (2003) showed in rodents an increased energy expenditure caused by catechins which was associated with an increase in protein content of BAT and that these effects were absent when the β-adrenoceptor was blocked. Thus, there is a possibility that a daily intake of catechin-rich beverages could increase BAT density in humans.

In the current study, we used NIR_TRS_ to examine the effects of prolonged catechin-rich beverage intake on BAT density and a potential association between changes in BAT density and local and whole-body adiposity in humans.

## Methods

### Study design

This study was conducted from December 2013 to March 2014, the winter season in Japan. Regional average air temperature and actual daytime duration at the time of measurement were 6.1–8.3 °C and 4.1–5.5 h, respectively. The subjects were given either catechin-rich (540 mg/day; catechin group) or placebo (placebo group) beverages every day for 12 consecutive weeks in a double-blind design.

We measured following parameters for participants in both groups at baseline and after 12 weeks of intervention during the luteal phase predicted from menstrual cycle and last menstrual period: BAT density, body composition (fat mass, percent body fat, lean body mass, and bone mass), visceral fat area (VFA), subcutaneous fat area (SFA), skinfold thickness, circulatory parameters [systolic blood pressure (SBP), diastolic blood pressure (DBP), and heart rate (HR)], and intramyocellular lipid (IMCL) and extramyocellular lipid (EMCL) concentrations. In addition, BAT density also measured at weeks 4, 6, 8, and 10. The subjects were instructed to maintain their usual dietary intake and physical activity. After obtaining all variables throughout the study period, the groups were unblinded before the variables were evaluated. The study design and protocol were approved by the Institutional Review Board of Ritsumeikan University, in accordance with the ethical principles contained in the Declaration of Helsinki. Written informed consent was obtained from all participants. This trial has been registered with the University Hospital Medical Information Network UMIN000019920.

### Participants

We recruited 22 healthy female college students by advertising on posters or by direct contact. Students taking any medications were excluded. The participants were randomly allocated to the catechin group or the placebo group by a third partly who did not participate in this study.

### Study sample

We used a commercially available catechin-rich beverage. Each plastic (350 mL) of the catechin-rich beverage contained 540.0 mg catechin, whereas bottles of the placebo beverage contained 0 mg catechin. The bottles were masked by using opaque plastic to make them indistinguishable in appearance. The caffeine content per bottle was 80.0 and 45.5 mg in the catechin-rich and placebo beverages, respectively (Table 1). Catechin, catechin gallate, gallocatechin, gallocatechin gallate, epicatechin, epicatechin gallate, epigallocatechin, and epigallocatechin gallate, and caffeine contents in the catechin-rich beverages were similar to those used in the previous study (Nagao et al. 2009). The timing for ingestion of the test beverage was not specified.

**Table 1:** Components of the test beverages

	Catechin-rich beverage	Placebo beverage
Total catechin (mg)	540	0
Caffeine (mg)	80	45.5
Total energy (kJ)	0	0
Total protein (g)	0	0
Total fat (g)	0	0
Carbohydrate (g)	3.9	0
Sodium (mg)	35	35

### Outcomes

The primary endpoint was BAT density evaluated by [total-Hb] using NIR_TRS_. The secondary endpoint was the changes in anthropometric parameters (body composition, fat thickness, and IMCL or EMCL concentration).

### Near-infrared time-resolved spectroscopy

The [total-Hb] was measured using NIR_TRS_ (TRS-20; Hamamatsu Photonics K.K., Hamamatsu, Japan) for 5 min at 27 °C by placing the probes on the skin in the supraclavicular region potentially containing BAT deposits and, as a reference, also in the deltoid muscle region, which is separated from the BAT deposits. The distance between the emitter and detector was set at 30 mm (Nirengi et al. 2015).

The tissue was illuminated using a 200-μm core diameter optical fibre by pulsed light generated from picosecond light pulsers at 760, 800, and 830 nm with 100-ps full width at half-maximum, a 5-MHz repetition rate, and an 80-μW average power of each wavelength. The emitted photons penetrated the tissue and were reflected to a 3-mm diameter optical bundle fibre through which they were sent to a photomultiplier tube for single photon detection and a signal processing circuit for time-resolved measurement. Using the non-linear least-squares method, the digitized temporal profile data from an in vitro sample or tissue was fitted with a theoretical temporal profile derived from the analytical solution of photon diffusion theory with a semi-infinite homogeneous model in reflectance mode, convoluted with the instrumental response function so the time response of the instrument itself could be compensated, and the values for absorption coefficient and reduced scattering coefficient at 760, 800, and 830 nm were obtained. Then, the absolute concentrations of [total-Hb] were determined using a least-squares fitting method (Hamaoka et al. 2007). The NIR_TRS_ system provided data every 10 s. The coefficient of variation for repeated measurements of [total-Hb] was 4.9 % (Nirengi et al. 2015).

### Anthropometric measurements

The body mass, fat mass, percent body fat, fat-free mass, and bone mass were evaluated by a dual-energy x-ray absorptiometry scan (DXA, Lunar Prodigy; GE Healthcare, Buckinghamshire, UK). The VFA and SFA at the abdominal level of L4–L5 were estimated using 1.5-T magnetic resonance imaging (MRI) (Signa HDxt; GE Healthcare, Buckinghamshire, UK). During DXA measurements, subjects maintained a supine position. Then, a series of transaxial MRI scans of abdominal sections were acquired [field of view 420 × 420 mm, slice thickness 10 mm, echo time (TE) = 7.3 ms, repetition time (TR) = one respiration]. The images were exported and analysed by the same investigator using image analysis software (SliceOmatic 4.3; Tomovision Inc, Magog, Canada).

### Subcutaneous fat thickness

B-mode ultrasonographic (SSD-3500SV; Hitachi Aloka Medical Co., Ltd, Tokyo, Japan) subcutaneous fat thickness was measured at the supraclavicular region potentially containing BAT and the deltoid muscle region which is separated from BAT deposits. During ultrasonographic measurements, subjects maintained the same posture as during the NIR_TRS_ measurement (Nirengi et al. 2016).

### IMCL and EMCL concentrations

IMCL and EMCL were measured in the vastus lateralis muscle by a 1.5-T proton magnetic resonance spectroscopy (^1^H-MRS) system (Signa HDxt; GE Healthcare, Buckinghamshire, UK) with an 8-channel body array coil positioned parallel to the main magnetic field. Multislice T1-weighted axial spin-echo images (TR/TE 600/7 × 8 ms, thickness 1 cm, field of view 44 cm, matrix size 512 × 512) were first acquired to guide the positioning of the volume of interest. Thereafter, single-voxel ^1^H-MRS measurements were made on the right vastus lateralis muscle at the midpoint between the greater trochanter and the knee cleft using the point-resolved spectroscopy sequence (TR/TE 2000/35 ms, 2 × 2 × 2 cm^3^, 32 acquisitions). The IMCL and EMCL concentrations were derived from the peak areas of the CH_2_ resonance and are expressed as ratios relative to the unsuppressed water peak area in the same voxel (Mayer et al. 2009). The peak chemical shifts of IMCL and EMCL were adjusted as 1.3 and 1.5 ppm, respectively.

### Circulatory parameters

SBP, DBP, and, HR were measured using an automated sphygmomanometer (HBP-9020; Omron Corp., Kyoto, Japan) after resting for 10 min.

### Dietary diary and records of intakes

Dietary habits during the preceding month were assessed using a validated brief self-administered diet history questionnaire that contained questions about the consumption frequency of 56 foods and beverages and nine dishes that are commonly consumed in the general Japanese population. Daily intakes of energy, protein, fat, and carbohydrate were calculated at baseline and after 12 weeks (Sugawara et al. 2014).

Daily steps and activity energy expenditure were estimated using pedometers (Omron Health Counter HJ-710IT; Omron Healthcare, Kyoto, Japan), and the mean for the 7 days was evaluated.

### Statistical analyses

Data are expressed as mean ± standard deviation (SD). Univariate regression analyses were used to analyse the relationship between changes in BAT density and the EMCL. A two-way analysis of variance with repeated measures was used to test the interaction (group × time) and main effect (group, time). If there was a significant interaction or main effect, the time or group differences of the variables between baseline and after 12-week were analysed using the Paired and unpaired *t* test, respectively. Values were considered to be statistically significant if *P* was <0.05. All statistical analyses were performed using SPSS version 19 (Chicago, IL, USA).

### Power calculation

A sample of 22 subjects was calculated based on detecting a difference of BAT density at the 12-week follow-up between the intervention and placebo groups, with 80 % power and 5 % significance. The difference (plus SD) was based on a previous study that examined changes in BAT activity with cold exposure in healthy subjects (Yoneshiro et al. 2013). The sample size was calculated using Easy R software (Saitama Medical Center, Jichi Medical University, Saitama, Japan) (Kanda 2013).

## Results

We recruited 22 healthy female college students [20.7 ± 2.0 years (mean ± SD), body mass index (BMI) 21.0 ± 1.4 kg/m^2^]. The subjects were given either a catechin-rich beverage (540 mg/day; catechin group; n = 11) or placebo beverage (placebo group; n = 11) every day for 12 consecutive weeks. One subject was excluded from the catechin group because of influenza. Therefore, data were analysed for 21 participants (Table 2). All anthropometric parameters and blood pressure showed no significant differences between catechin and control groups at baseline or at the end of the 12-week study period (Table 2). There were no significant changes in the physical activity levels and dietary intakes during the intervention (data not shown).

**Table 2:** Changes in anthropometric parameters and blood pressure between baseline and after 12 weeks intervention in the catechin and placebo groups

Variables	Catechin (n = 10)		Placebo (n = 11)	
	Baseline	12 weeks	Baseline	12 weeks
Age (years)	21.1 ± 2.0	21.1 ± 2.0	20.5 ± 2.1	20.5 ± 2.1
Body weight (kg)	55.9 ± 4.2	56.5 ± 4.4	55.7 ± 6.0	55.8 ± 7.8
BMI (kg/m^2^)	21.1 ± 1.3	21.2 ± 1.5	20.9 ± 1.6	20.9 ± 1.9
Body fat content (%)	24.0 ± 3.5	24.8 ± 2.9	25.8 ± 7.6	26.0 ± 8.7
Lean body mass (kg)	40.5 ± 3.8	40.6 ± 3.6	38.3 ± 8.2	38.2 ± 8.3
Body fat mass (kg)	12.8 ± 2.1	13.4 ± 1.8	14.0 ± 5.3	14.3 ± 6.3
Bone mass (kg)	2.6 ± 0.2	2.6 ± 0.3	2.4 ± 0.5	2.4 ± 0.5
VFA (cm^2^)	27.6 ± 7.3	29.3 ± 11.0	30.8 ± 13.2	34.2 ± 17.2
SFA (cm^2^)	117.2 ± 30.8	121.6 ± 29.5	124.1 ± 59.5	136.3 ± 69.0
Supraclavicular subcutaneous fat thickness (cm)	0.22 ± 0.02	0.23 ± 0.03	0.23 ± 0.2	0.24 ± 0.02
Deltoid muscle subcutaneous fat thickness (cm)	0.38 ± 0.06	0.36 ± 0.04	0.40 ± 0.01	0.41 ± 0.10
SBP (mm Hg)	117 ± 7	112 ± 9	112 ± 7	107 ± 8
DBP (mm Hg)	66 ± 7	66 ± 9	59 ± 6	61 ± 8
Heart rate (bpm)	69 ± 10	74 ± 7	67 ± 8	69 ± 8

*BMI* body mass index, *VFA* visceral fat area, *SFA* subcutaneous fat area, *SBP* systolic blood pressure, *DBP* diastolic blood pressure

**Fig. 1:**
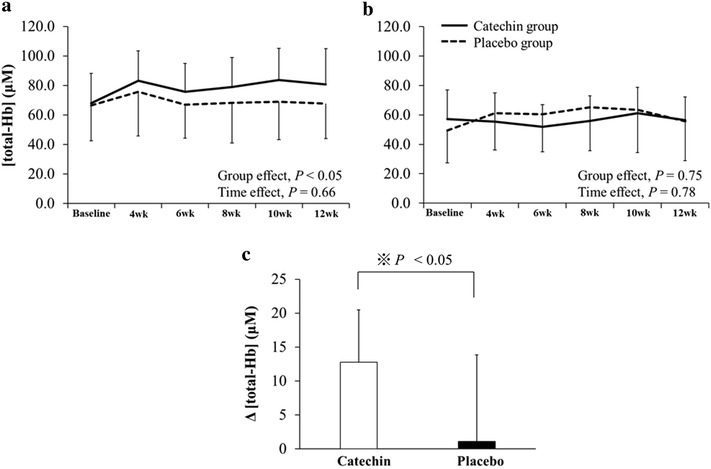
**a**, **b** Changes in total haemoglobin concentration [total-Hb] in **a** the supraclavicular region potentially containing brown adipose tissue and **b** deltoid muscle region separated from brown adipose tissue deposits. **c** Changes in [total-Hb] before and after the intervention

There was a significant main group effect on the [total-Hb] in the supraclavicular region (Fig. 1a) but not in the deltoid muscle region, which is separated from the BAT depots (Fig. 1b). There was a significant increase in [total-Hb] between baseline and after 12 weeks in the catechin group (67.9 ± 20.4 vs. 80.6 ± 24.3 μM; *P* < 0.01), while there was no change in the placebo group (66.6 ± 24.0 vs. 67.7 ± 23.7 μM; *P* = 0.78). Further, the change in the [total-Hb] in the supraclavicular region during the 12-week period was significantly higher in the catechin group than in the placebo group (Fig. 1c).

**Fig. 2:**
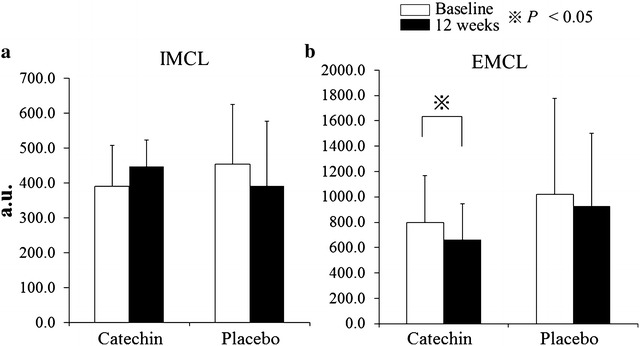
**a**, **b** Effect of 12 weeks of catechin or placebo beverage ingestion on the (**a**) extramyocellular lipid concentration (EMCL) and **b** intramyocellular lipid (IMCL) concentration. The extramyocellular lipid concentration was significantly decreased in the catechin group after 12 weeks of catechin-rich beverage ingestion (*P* < 0.05). *a.u.* arbitrary units

There was a significant interaction effect (time × treatment group) on the EMCL concentration, which was significantly decreased by 17.4 % catechin group. There was no significant change in EMCL concentration in the placebo group (Fig. 2a). The IMCL concentration did not change significantly in either the catechin or the placebo group (Fig. 2b).

**Fig. 3:**
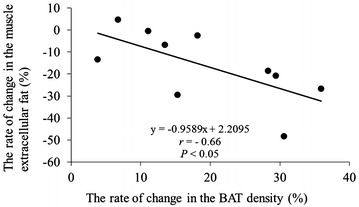
There was a significant negative correlation between the changes in brown adipose tissue (BAT) density evaluated by total haemoglobin concentration [total-Hb] and extramyocellular lipid concentration after catechin-rich beverage ingestion (*r* = −0.66, *P* < 0.05)

A negative correlation was found between the changes in BAT density and EMCL concentration (*r* = −0.66, *P* < 0.05) (Fig. 3).

No apparent harmful incidents were observed in any individuals in the present study.

## Discussion

To the best of our knowledge, this is the first study to examine whether a catechin-rich beverage increases BAT density in humans. In this study, the BAT density as evaluated by the [total-Hb] was increased (19 %) only in the catechin group in the supraclavicular region, whereas it did not change in the deltoid muscle of both groups after 12 weeks.

It is reported that there is a considerable intra-individual variance in BAT density, which is even true using a controlled protocol such as chronic cold exposure study (Hanssen et al. 2015; Blondin et al. 2014; van der Lans et al. 2013; Yoneshiro et al. 2013). Thus, the possibility of the type I and II error due to the intra-individual variance in BAT density could influence the results of this study.

It is known that catechins inhibit the catecholamine-degrading enzyme catechol-*O*-methyltransferease (COMT) in vitro, which results in the sustained effect of NE and thereby the increased lipolysis and BAT activation (Hursel and Westerterp-Plantenga 2013) Catechin- and caffeine-rich teas for control of body weight in humans (Hursel and Westerterp-Plantenga 2013). However, Lorenz et al. (2014) recently reported in humans that COMT activity is not inhibited by high doses of EGCG, indicating a negligible role of COMT in the catechin effects in vivo. Alternatively, an increase in the amount of BAT or activation of BAT is mainly regulated by the increased sympathetic outflow from the hypothalamus to the BAT. It is reported that stimulation of transient receptor potential (TRP) channels is effective for enhancement of BAT thermogenesis and upregulation of UCP-1, a key molecule of BAT thermogenesis (Morrison et al. 2014; Ono et al. 2011). This pathway has been extensively studied in other functional ingredients such as capsinoids in human as well as animals (Saito and Yoneshiro 2013). It was reported that catechin activates TRP channels in cell cultures of taste sensory cells from the gut (Kurogi et al. 2012). Therefore, it is speculated that increasing BAT density found in this study might be due to the gut TRP channel activation. Further investigation is needed to elucidate the detailed mechanism for the increase in BAT density by a catechin-rich beverage intake.

Several different strategies to increase BAT activity and overall BAT mass have been attempted by various groups (Hanssen et al. 2015; Blondin et al. 2014; van der Lans et al. 2013; Cypess et al. 2015; Yoneshiro et al. 2013). These strategies include cold acclimation (Hanssen et al. 2015; Yoneshiro et al. 2013), and repeated functional foods (Nirengi et al. 2016; Yoneshiro et al. 2013; Saito and Yoneshiro 2013) which have both been shown to increase energy expenditure through BAT activation. Acute pharmaceutical drug treatment, such as β_3_-adrenergic receptor (AR) agonists (Cypess et al. 2015) and bile acid (Broeders et al. 2015), have also been used to increase BAT activity. Cold exposure would be difficult to incorporate into daily life (Yoneshiro et al. 2013) and β_3_-AR may cause adverse side effects, such as increased blood pressure (Cypess et al. 2015). On the other hand, functional foods have no apparent side effects, unlike pharmaceuticals, and they are easy to incorporate into daily life. Therefore, we used commercially available catechin-rich beverage, green tea, which has the highest catechin concentration in Japan and is approved by the FDA. In addition, this beverage have used in many studies (Nagao et al. 2007, 2009; Hase et al. 2001). The differential caffeine content between catechin and placebo groups is a confounder. Caffeine enhances BAT activity by upregulating intracellular cyclic AMP or by sympathetically releasing NE in vitro (Dulloo et al. 2000). However, a previous study reported that the intake of caffeine (10 mg/kg of body weight), which is approximately 7- to 11-fold of the amount used in the present human study, did not increase FDG uptake in the BAT in rats (Baba et al. 2007). In addition, a tolerance develops to the effects of caffeine (250 mg) on plasma and urinary catecholamines at day 3 in humans (Robertson et al. 1981). Thus, it suggested that caffeine did not influence our results. Nevertheless, we highlight a new practical method for increasing BAT mass by using catechin-rich beverage regardless of the presence or absence of caffeine. However, the increase in BAT mass or activity in observed herein was smaller than that in previous cold exposure studies (37–58 %) (Blondin et al. 2014; van der Lans et al. 2013; Yoneshiro et al. 2013). Further studies are needed to explore functional foods, which are less effective in increasing BAT density compared with cold acclimation.

Previous studies have reported that ingesting a catechin-rich beverage decreased VFA and SFA in obese (BMI 24–30 kg/m^2^) humans (Nagao et al. 2007) and decreased the waist circumference of patients with type 2 diabetes mellitus (Nagao et al. 2009). Thus, these studies show that catechin decreased body fat. Meanwhile, some studies have reported that there was no significant decrease in body fat after catechin intake (Fukino et al. 2005; Leenen et al. 1992). Hase et al. (2001) reported that subjects with BMI ≥ 25 showed a decrease in body fat, but subjects with BMI < 25 showed no changes in body fat during 12 weeks of catechin ingestion. Although the amount of ingestion and the study period were similar to previous studies (Nagao et al. 2007, 2009), the decrease in body fat was lower in subjects with lower body fatness than in those with higher body fatness (Leenen et al. 1992). Subjects in our study had low BMI (average 21.0 ± 1.4 kg/m^2^), which is one possible reason why we observed no changes in the whole-body fat parameters despite the increase in BAT density.

In this study, 12 weeks of catechin-rich beverage ingestion led to a significant decrease in EMCL. EMCL has been related to a decrease in insulin sensitivity (Hausman et al. 2014) and an increase in arterial stiffness (Hasegawa et al. 2015), whereas the role of IMCL is as an energy source, such as during acute exercise (Rico-Sanz et al. 2000). Dulloo et al. (1999) reported that treatment with green tea extract resulted in a significant increase in 24-h energy expenditure and a significant decrease in the 24-h respiratory quotient. An increase in energy expenditure may be associated with an increase in β-oxidation in liver (Murase et al. 2002) and muscle (Murase et al. 2006) after catechin ingestion. The molecular mechanism of lipid oxidation from catechin ingestion may, at least in part, be activation of liver kinase B1/AMP-activated protein kinase in various tissues (Murase et al. 2009). Therefore, we considered that an increase in muscle β-oxidation induced by catechin intake may, at least in part, account for the decrease in EMCL. We also speculated in a recent study that in normal cells, catechin might selectively affect the activity of sirtuin 3 (Tao et al. 2015), which regulates mitochondrial fatty acid oxidation (Hirschey et al. 2010). In this study, there was a significant negative correlation between the changes in BAT density and the changes in EMCL. Although there might be a coincident relationship between changes in BAT density and EMCL after 12 weeks intervention and a common pathway might simultaneously activate the two factors, the possibility cannot be ruled out that EMCL decreased due to an unidentified hormonal factor released from the BAT deposits. To our knowledge, this is the first study in which the decrease in EMCL was associated by catechin ingestion. Further studies are required to understand why EMCL showed a decreased response.

There were several limitations in this study. NIR_TRS_, which is an indirect measurement of BAT density, cannot be used to evaluate the responsiveness of BAT activity to an acute metabolic change such as an acute cold exposure in nature because NIR_TRS_ does not reflect the blood flow, but the blood volume. According to our previous study (Nirengi et al. 2015), the magnitude of the increase in blood volume detectable by NIR_TRS_ is presumably much smaller than that of blood flow velocity during highly metabolic activity. It remains a challenge for future research to confirm validation using ^18^FDG-PET/CT experiments and monitor energy expenditure such as cold-induced thermogenesis. In this study, we conducted an experiment with a double-blind design. Although some subjects, who may have previously consumed a catechin-rich beverage similar to the one used in this study, might identify the type of beverage based on its taste, we do not believe that knowing its type influenced the physiological consequences. Nonetheless, a study using taste-blind capsules is needed to eliminate completely a placebo effect. Only young female college students were included in this study. Therefore, the effect of catechin-rich beverage ingestion on obese or elderly individuals with a lower BAT content remains to be elucidated.

## Conclusions

In conclusion, our results indicate that repeated ingestion of a catechin-rich beverage increases BAT density evaluated by NIR_TRS_ method with a concomitant decrease in EMCL. Although the degree of increasing in BAT mass was smaller than previous cold exposure studies, the result of this study widens the choice of methods for increasing BAT activity/mass. Daily ingestion of a catechin-rich beverage may be useful as an easier and more convenient treatment than chronic cold exposure once the effectiveness for the increase in BAT. ^18^FDG-PET/CT experiments is required to extensively support the conclusion of the study.

## Abbreviations

AMP: adenosine monophosphate
BAT: brown adipose tissue
BMI: body mass index
DBP: diastolic blood pressure
DXA: dual-energy X-ray
TE: echo time
EMCL: extramyocellular lipid
FDG-PET/CT: ^18^F-fluorodeoxyglucose positron emission tomography combined with computed tomography
HR: heart rate
IMCL: intramyocellular lipid
MRI: magnetic resonance imaging
NA: noradrenaline
NIR_TRS_: near-infrared time-resolved spectroscopy
^1^H-MRS: proton magnetic resonance spectroscopy
TR: repetition time
SFA: subcutaneous fat area
SBP: systolic blood pressure
SNS: sympathetic nervous system
[total-Hb]: total haemoglobin concentration
TRP: transient receptor potential channels
UCP-1: uncoupling protein 1
VFA: visceral fat area
